# Familiar Forms of Nervous Disease

**Published:** 1890-12

**Authors:** 


Familiar Forms of Nervous Disease. By M. Allen Starr M. D., Ph. D. Pp. 330, William Wood & Co., New York, 1890.

This work is the embodiment of a series of clinical studies of some forms, though scarcely of the more familiar forms of nervous disease, based upon the analyses of numerous cases selected from an abundant supply of clinical material at the disposal of the accomplished author.

The larger part of the work is devoted to a consideration of the localization of cerebral functions, of the affections of the motor and the visual areas, of aphasia, and of the localization of spinal cord disorders. Then follow short chapters on locomotor ataxia, infantile paralyses, multiple neuritis, paralysis agitans, chorea, epilepsy, neurasthenia and epilepsy.

The last chapters are devoted, respectively, to electricity as a therapeutic agent, and to formulæ in common use in this class of diseases.

In reviewing the facts pertaining to the employment of electricity, the author says: "I cannot, however, close this chapter without stating that after the constant use of electrical treatment for the past six years, in dispensary and in private practice, I have been disappointed in the results obtained. * * My experience coincides with that of Gowers, that the therapeutic effects of electrical applications have been much exaggerated and are really quite limited.

The object of the writer in preparing this volume, has been to place in the hands of the general practitioner the available results of recent researches and discoveries in this department of medicine. Results which render the diagnosis of otherwise obscure affections easier and more exact, thus opening the way to the successful medical or surgical treatment of diseases heretofore regarded, in large measure, as beyond the reach of direct remedial agents.

The book is entertaining throughout. It is written in an easy and attractive style. The text, though concise, is sufficiently full and clear and is amply and well illustrated. The pathological and the therapeutical views enunciated are in accord with the accepted and the most advanced knowledge of the time.

The little volume deserves the attention and the confidence of the medical profession. J. B. B.

